# Supplementary material for: Assessing Regional Weather’s Impact on Spinal Cord Injury Survivors, Caregivers, and General Public in Miami, Florida
Source: Int J Environ Res Public Health. 2024 Mar 22;21(4):382. doi: 10.3390/ijerph21040382 (PMC11050493; doi:10.3390/ijerph21040382)
Supplement: Supplementary file 1 [file ijerph-21-00382-s001.zip › ijerph-2898408-supplementary.pdf]

International Journal of
Environmental Research and Public Health

MDPI

Supplemental Table S1. Binomial Regression Summary Results.

Commute Interruptions				
Coefficients:				
	Estimate	Std. Error	z value	Pr(>|z|)
(Intercept)	−1.89797	0.41527	−4.57	4.87e-06 ***
Extreme Heat: A lot	−0.02345	0.71115	−0.033	0.9737
Extreme Heat: Somewhat	0.64397	0.71673	0.898	0.3689
Flood: A lot	2.00259	1.03945	1.927	0.0540
Flood: Somewhat	1.27988	0.6776	1.889	0.0589
Heavy: Rain A lot	0.80385	0.84544	0.951	0.3417
Heavy: Rain Somewhat	0.4188	0.65762	0.637	0.5242
**Attendance**				
Coefficients:				
	Estimate	Std. Error	z value	Pr(>|z|)
(Intercept)	−2.0411	0.5702	−3.58	0.000344 ***
Extreme Heat: Reschedule	−2.179	1.1321	−1.925	0.054267
Extreme Heat: Cancel/ no show	−2.3648	1.478	−1.6	0.109602
Flood: Reschedule	0.6566	0.6682	0.983	0.325772
Flood: Cancel/ no show	1.3782	0.9695	1.422	0.15517
Heavy Rain: Reschedule	1.2422	0.5693	2.182	0.029114 *
Heavy Rain: Cancel/ no show	1.6572	1.3665	1.213	0.225236

***: 0.001; **: 0.01; *: 0.05.
